# Healthier without healthcare? The paradox of the common cold

**DOI:** 10.1186/s12931-018-0964-z

**Published:** 2018-12-27

**Authors:** Daniele de Luca, Oliver Schildgen

**Affiliations:** 1 Division of Pediatrics and Neonatal Critical Care, Medical center „A.beclere“, South Paris University Hospitals, APHP, Paris, France; 2 Dipl.-Biologe, Fachvirologe GfV Institut für Pathologie, Kliniken der Stadt Köln gGmbH, Klinikum der Privaten Universität Witten/Herdecke, Ostmerheimer Str. 200, D-51109 Cologne, Germany

## Editorial

The common cold remains a serious clinical problem worldwide that causes severe economic burden and objectively and subjectively disturbs the individual patient’s wellbeing. In the majority of cases, the disease is mild and self-limiting, but for patients with underlying chronical diseases also the common cold can become life-threatening. However, the rest of the patients, i.e. the vast majority of common cold patients, are likely to experience an acute but often harmless clinical course. Anyhow, it is exactly this majority of patients who is accessing massive treatments, which is, in the best case, solely symptomatically, but can also be represented by antibiotic therapy even in the absence of a bacterial pathogen. This seems especially common in the infancy and in the elderly, which are also the ages with higher incidence of comorbidities and more at risk of antibiotic-resistant infections [1, 2].

**Fig. 1:**
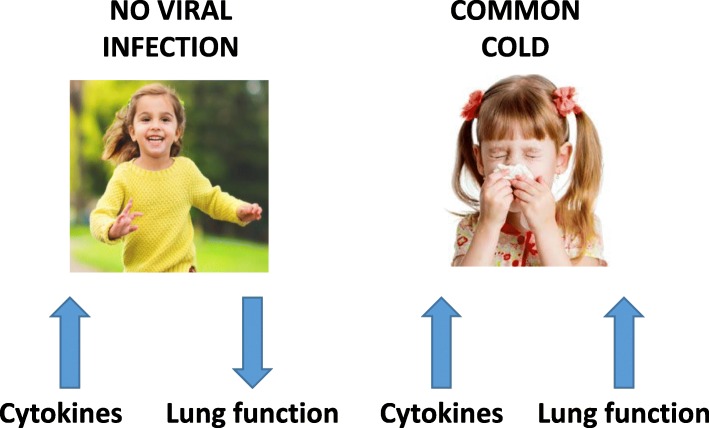
Without infection increased cytokine levels appear to reduce lung function, a phenomenon that turns around during a viral respiratory infection

With this background the recent study by Lewis and colleagues (Lewis et al., Respiratory Research, in press) may lead to a provoking conclusion. The authors have investigated the airway cytokine profiles and lung function in asthmatic children. They found that in the absence of viral infection, higher cytokine levels are associated with decreasing lung function. However, with infection, there is a reversal in this relationship, with cytokine abundance associated with reduced lung function decline (Fig. 1). Whilst the data do not show that viral infection reduce the burden of asthma, with the due limitations related to the sampling technique, these data suggest that some aspects of the inflammatory response may be protective against viral infection.

The provoking conclusion from the study could be - on a larger scale - that we should not necessarily try to lower inflammation during rhinitis and similar viral upper airway infections. From a public health perspective we know that rhinitis is probably the commonest disease ever, and from a sociological point of view, everybody (at least in western world) tends to overtreat and take a lot of (more or less useless) medications for this. These data support a healthier, wiser, evidence-based “wait and see” policy. Such a “wait and see” policy, however, should never lose sight of the fact that childhood infections with some viruses such as RSV and rhinoviruses remain a major risk for the development of asthma, could trigger asthma exacerbations, or could reduce the lung function in children [3–6]. Thus, any wait and see policy should be accompanied by proper molecular diagnostics in order to monitor the risk for asthma development.

## Conclusion

In the future it would instead be better to optimize and broader apply the molecular diagnostics, identifying the pathogens involved in the outpatients’ settings, and stratifying the patients in cohorts infected with asthma-inducing pathogens (such as Respiratory Syncytial Virus) or with pathogens inducing protective immune responses. In any case, it would make sense to extend studies on respiratory viruses to the non-hospitalized patients. In fact, despite a plethora of clinical studies on respiratory infections this cohort remains under-investigated and thus underrepresented in the available literature. These research steps will allow us to spare unnecessary therapy and to provide a more personalized approach to the commonest respiratory infections.
